# Highly Selective NH_3_ Sensor Based on MoS_2_/WS_2_ Heterojunction

**DOI:** 10.3390/nano13121835

**Published:** 2023-06-10

**Authors:** Min Zhang, Jinzhu Zhang

**Affiliations:** 1 Xinjiang Key Laboratory of Solid State Physics and Devices, Xinjiang University, Urumqi 830046, China; 18894001858@163.com; 2 School of Physics Science and Technology, Xinjiang University, Urumqi 830046, China

**Keywords:** MoS_2_/WS_2_, anti-humidity, ammonia sensing properties, heterojunction

## Abstract

In this paper, the heterostructure of MoS_2_/WS_2_ was prepared by a hydrothermal method; the n-n heterostructure was demonstrated using TEM combined with Mott-Schottky analysis. The valence and conduction band positions were further identified by the XPS valence band spectra. The NH_3_-sensing properties were assessed at room temperature by changing the mass ratio of the MoS_2_ and WS_2_ components. The 50 wt%-MoS_2_/WS_2_ sample exhibited the best performance, with a peak response of 23643% to NH_3_ at a concentration of 500 ppm, a minimum detection limit of 20 ppm, and a fast recovery time of 2.6 s. Furthermore, the composites-based sensors demonstrated an excellent humidity immune property with less than one order of magnitude in the humidity range of 11–95% RH, revealing the practical application value of these sensors. These results suggest that the MoS_2_/WS_2_ heterojunction is an intriguing candidate for fabricating NH_3_ sensors.

## 1. Introduction

Ammonia (NH_3_) is a colorless and strongly irritating odor gas that is among the most prevalent indoor pollutant gases that is lighter than air (specific gravity of 0.5), with a minimum perceptible concentration of 30 ppm [1]. The solubility of ammonia is so high that it has an irritating and corrosive effect, particularly on the upper respiratory tracts of animals or humans. Humans’ respiratory, pulmonary, and immune systems suffer severe damage when exposed to environments with NH_3_ concentrations above 50 ppm for more than 30 min [2,3]. Therefore, it is critical to effectively monitor low-concentration NH_3_ in real time.

When compared to semiconductor gas sensors, the methods typically employed for detecting gases are spectrophotometric [4], gas chromatography [5], and electrochemical sensors [6], but these have specific limitations, such as complicated operation, high costs, and inability to monitor in real-time, thereby restricting their practical applications. Semiconductor gas sensors are widely used in environmental monitoring and security systems due to their portability, simplicity of operation, and low detection costs [7,8].

Tungsten disulfide (WS_2_), a typical n-type semiconducting material, has been utilized in light-emitting diodes, energy storage devices, and semiconductor gas sensors [9,10]. WS_2_ with two-dimensional morphology generally exhibits a large specific surface area, high electron mobility, and excellent thermal stability. Shivani Sharma [11], and Xu [12] et al. reported that WS_2_ nanosheet-based ammonia (NH_3_) sensors exhibited an excellent performance. Li et al. [13] employed commercially available WS_2_ powder to construct a WS_2_ nanosheet-based NH_3_ sensor with a long response time. However, the response towards NH_3_ increased as the ambient humidity increased. The WS_2_ nanosheets were unstable due to the adsorption of water molecules in the air, and also their sensitivity to NH_3_ was deemed to be too low for practical applications [14,15,16,17,18,19,20]. Researchers have explored that the primary cause of this instability is weak van der Waals force interactions between the adjacent planes in WS_2_, which enable gas molecules to freely permeate and diffuse between the layers and ultimately cause changes in the conductivity of the WS_2_ material, which in turn induces the unstable gas-sensitive performance of gas sensors made from a pristine WS_2_ [21,22].

As a typical two-dimensional transition metal sulfide (TMD) material, MoS_2_ has been widely used in electrodes, energy storage devices, and catalysis [23,24,25]. Two-dimensional MoS_2_ is a direct bandgap semiconductor with an optical band gap of 1.8 eV [26], and both MoS_2_ and WS_2_ are common 2D TMD materials with excellent stability. It has previously been reported in the literature that compositing MoS_2_ with WS_2_ improves the unstable properties of WS_2_ as a sensitive substrate while also providing a high sensitivity. Their composites would integrate the benefits of both components and possess superior properties to any single one by producing a heterostructure between WS_2_ and MoS_2_ [27]. The high separation and transfer efficiency of carriers induced by the heterojunction is of great significance to the high response speed of the sensors.

In this study, we developed an NH_3_ sensor based on MoS_2_/WS_2_ heterostructures, which was previously demonstrated theoretically. The introduction of such heterostructures increase the baseline resistance of sensors, thus improving the sensing response at room temperature, which is of great significance for the real-time monitoring of ammonia gas. Then, the response behavior of MoS_2_/WS_2_ to NH_3_ was systematically investigated. As predicted, the gas sensor based on the MoS_2_/WS_2_ heterostructure exhibited an enhanced NH_3_-sensing performance, significantly lower detection limits, fast recovery, reliable resistance to moisture interference, and excellent stability. Thus, this work provides a practical approach for developing high-performance NH_3_ gas sensors at room temperature. 

## 2. Materials and Methods

### 2.1. Materials

Thioacetamide (C_2_H_5_NS), thiourea (CH_4_N_2_S), tungsten chloride (WCl_6_), and sodium molybdate (Na_2_MoO_4_·2H_2_O) were all purchased from Shanghai Maclean, Shanghai, China. Standard gases, HCHO, C_2_H_6_O, C_3_H_6_O, H_2_O_2_, and NH_3_ were purchased from Aladdin, Seattle, WA, USA. All reagents used in the experiments were of analytical grade.

### 2.2. Preparation of WS_2_

WS_2_ nanosheets were synthesized by a hydrothermal method. A total of 5 mmol of WCl_6_ and 25 mmol of C_2_H_5_NS were dissolved in 30 mL of deionized water, stirred magnetically for 1 h, transferred to a 50 mL autoclave, and kept at 160 °C for 10 h. The residuals were washed and centrifuged 3–5 times with deionized water and ethanol until a neutral pH was obtained and treated with ultrasound for 15 min. Finally, the products were treated in a vacuum drying oven at 60 °C for 24 h.

### 2.3. Preparation of MoS_2_

A total of 2.5 mmol of CH_4_N_2_S and 0.545 mmol of Na_2_MoO_4_·2H_2_O were weighed and mixed with 70 mL of deionized water. After that, the mixture was completely dissolved by magnetic stirring, transferred to an autoclave lined with PTFE and placed in a constant temperature oven at 200 °C for 12 h. Then, it was removed and cooled naturally. The reaction products were washed with deionized ethanol and water alternately, centrifuged, and dried at 70 °C for 10 h. Finally, MoS_2_ powders were obtained.

### 2.4. Preparation of MoS_2_/WS_2_ Composites 

The obtained MoS_2_ and WS_2_ samples were mixed at 30 wt%: 70 wt%, 50 wt%: 50 wt%, and 70 wt%: 30 wt%, respectively, with 50 mL of deionized water, and these solutions were added to an autoclave lined with PTFE. The autoclave was kept at 200 °C for 10 h, cooled to room temperature, and washed several times with ethanol and water. Finally, it was vacuum dried at 60 °C for 10 h to obtain MoS_2_/WS_2_ nanocomposites containing different relative mass ratios, which were later named as 3 MW, 5 MW, and 7 MW, respectively.

### 2.5. Characterization

The crystal structure of the prepared sensing materials was analyzed using X-ray diffraction (XRD, D8 Advance, Cu-Kα) at an operating voltage of 60 kV. To assess the Raman spectra of the prepared samples in the range of 200–700 cm^−1^, a Raman microscope with a 532 nm laser source was used. The band-gap of the samples was tested using a UV-visible spectrophotometer (UV-visible spectrophotometer, Lambda 650, PerkinElmer, Waltham, MA, USA). X-ray photoelectron spectroscopy (XPS) analysis was performed using a ThermoEscalab 250 (Thermo Fisher Scientific, Waltham, MA, USA) equipped with a monochromatic Al Ka source (hv = 1486.6 eV) to collect the formation of surface elemental assemblages and elemental states. The morphology and elements of the products were observed using a field emission scanning electron microscope (SEM, Apreo S, FEI, Hillsboro, OR, USA) equipped with an energy-dispersive X-ray spectrometer (EDX, Bruker, German). The internal microstructure of the material was observed with high resolution transmission electron microscopy (HR-TEM, FEI-Tecnai G2T20, FEI Company, Hillsboro, OR, USA). The surface functional groups of the samples were evaluated using FTIR spectroscopy (Bruker, Billerica, MA, USA) in the wavelength range of 500–4000 cm^−1^. The capacitance-voltage (C-V) relationship curves of the samples were measured with an electrochemical workstation (CHI660E model) and valence band XPS tests were performed using a Thermo Scientific ESCALAB Xi^+^ multifunctional electron spectrometer.

### 2.6. Gas Detection

First, the prepared WS_2_, MoS_2_ and MoS_2_/WS_2_ samples were placed in a mortar and then a small amount of deionized water was added and ground for 3 min. The Ag-Pd electrode with the ceramic substrate was then adopted, being 13.4 mm long and 7 mm wide, with a minimum width and spacing of 0.2 mm. After grinding, they were pasted on the Ag-Pd electrode sheet with a small brush and dried in a vacuum drying oven at 50 °C for 10 h. After drying, the gas-sensitive characteristics of all the samples were tested at 1 V using a comprehensive photoelectric test bench (CGS-MT). The level of the sensing response of the sensor is determined by the relative value of the current change in gas and in air, i.e., R = (I − I_0_)/I_0_, where I_0_ is the current of the device in the air, and I is the current in the gas atmosphere to be measured. When the device is transferred from air to the gas to be detected, the time required for the current to reach a steady state is the response time, and when the device is removed from the gas to the air, the time required for the current to reach a steady state is determined as the recovery time.

### 2.7. Moisture Resistance Measurement

**Figure 1:**
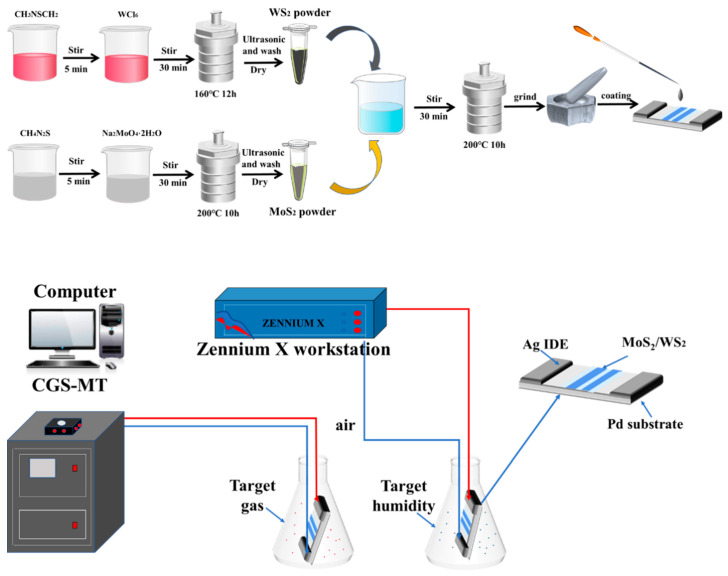
The manufacturing process and testing process of the WS_2_/MoS_2_ sensor.

In moisture resistance testing, different relative humidity environments are obtained by formulating different saturated salt solutions. Different humidity levels (11–95% RH) were obtained by preparing LiCl, MgCl_2_, K_2_CO_3_, Mg(NO_3_)_2_, CuCl_2_, NaCl, KCl, and KNO_3_ saturated salt solutions with the relative humidity of 11%, 33%, 43%, 54%, 64%, 75%, 85%, and 95%, respectively. The air humidity was 30%RH at the time of testing, with the test voltage set at AC 1 V, and a frequency of 100 Hz, respectively. The device was sequentially placed into chambers of different relative humidity for 6 min to obtain the resistance response. The manufacturing process and testing process of the WS_2_/MoS_2_ sensor was shown in Figure 1. 

## 3. Results

**Figure 2:**
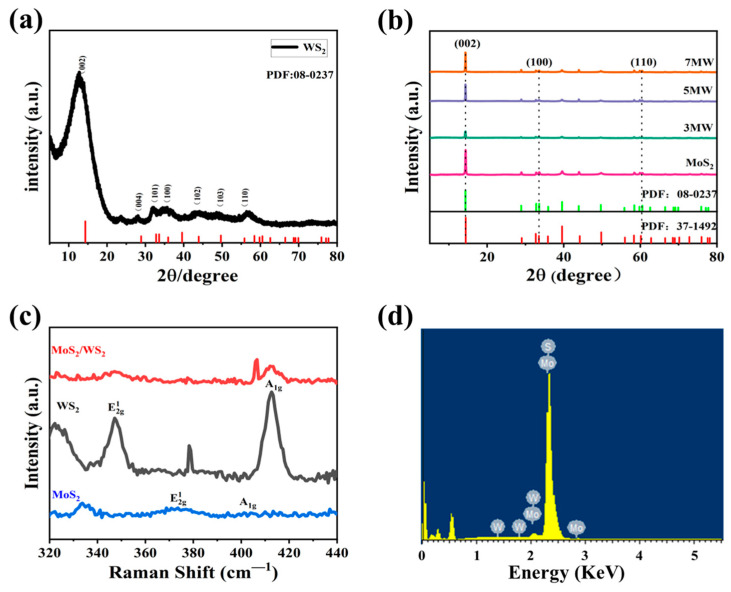
(**a**,**b**) The XRD patterns of the prepared WS_2_, MoS_2_, and MoS_2_ /WS_2_ composites. (**c**) The Raman spectra of WS_2_, MoS_2_, and 5 MW. (**d**) The corresponding energy dispersive X-ray spectrum (EDS) of the 5 MW sample.

Figure 2a,b illustrates the XRD analysis of the crystal structures of WS_2_ and MoS_2_, where 2θ is the diffraction angle. The spectrum of WS_2_ exhibits more distinct characteristic peaks at approximately 14.44° (002), 32.77° (100), 33.58° (101), 43.90° (103), and 58.42° (110), respectively, indicating that the WS_2_ sample belongs to 2H-WS_2_ (JCPDS:08-0237) [28]. The wide full width at half maxima and the weak peak intensity of WS_2_ implies the poor crystallinity of the sample. MoS_2_ peaks were located at 59.4° for the (110) plane, 32.7° for the (100) plane, and 14.44° for the (002) plane, respectively, corresponding to the 2H phase MoS_2_ (JCPDS:37-1492) [29] with good crystallinity. Furthermore, the MoS_2_/WS_2_ composites were analyzed through XRD analysis. In the composite samples, three distinct peaks were observed; the diffraction peak of the (002) (100) (110) crystal planes originated from MoS_2_, and (101) (103) (110) from WS_2_, respectively. This analysis demonstrated that the phase composition of the composite samples is a mixture of WS_2_ and MoS_2_ [30]. It has been observed that the peaks of the (002) crystal plane in the MoS_2_/WS_2_ composites exhibited a high intensity. The diffraction peak intensity of the composites gradually increased as the MoS_2_ composition increased, and the full width at half maxima narrowed, indicating that crystallinity improved as the relative concentration of MoS_2_ in the composites increased. At the same time, no other impurity peaks were detected in the XRD patterns, revealing that WS_2_ and MoS_2_ are chemically compatible [31].

Figure 2c depicts the Raman spectra of WS_2_, MoS_2_, and 5 MW samples. Both WS_2_ and MoS_2_ have a hexagonal structure [32], and two main Raman resonance modes: E^1^_2g_ (in-plane phonons) and A_1g_ (out-of-plane phonons). The resonance signals at 350.4, and 418.8 cm^−1^ in WS_2_ correspond to the E^1^_2g_ and A_1g_ modes, whereas the signals at 376.6 cm^−1^ and 404.8 cm^−1^ in MoS_2_ correspond to the E^1^_2g_ and A_1g_ modes, respectively. The XRD analysis and Raman spectra of the WS_2_ and MoS_2_ resonance signals confirmed the successful synthesis of the MoS_2_/WS_2_ composites [33]. The energy dispersive spectral analysis of the 5 MW sample depicted in Figure 2d reveals the presence of the elements S, Mo, and W.

**Figure 3:**
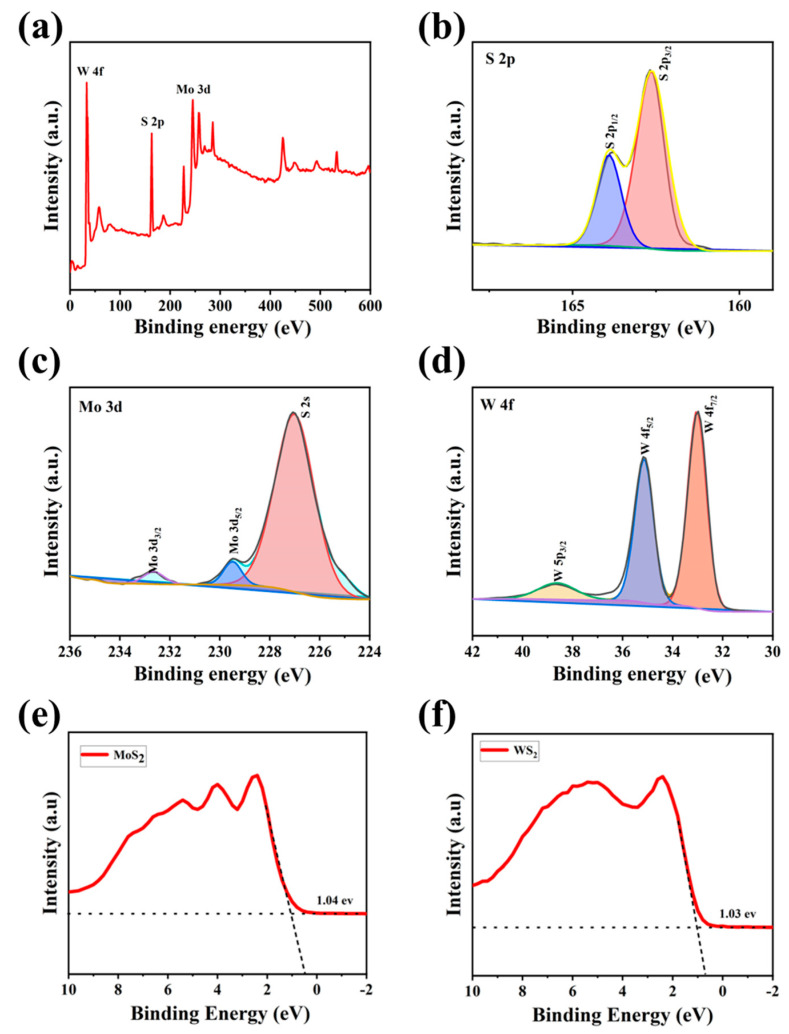
The XPS spectra of the 5 MW sample: (**a**) the survey spectrum, (**b**) high-resolution S 2p, (**c**) high-resolution Mo 3d, (**d**) high-resolution W 4f of the 5 MW sample, and (**e**,**f**) the VB-XPS spectra of MoS_2_ and WS_2_.

The surface composition and chemical state of the MoS_2_/WS_2_ composite were determined using XPS analysis, and the results are shown in Figure 3. We used the C 1s peak of 284.8 eV as a reference for the binding energy. The characteristic peaks of S 2p, Mo 3d, Mo 3p, and W 4f were observed in the MoS_2_/WS_2_ composites from the broad scan spectrum (Figure 3a). High-resolution XPS spectra were employed to scan the orbital peaks of Mo 3d, W 4f, and S 2p, as demonstrated in Figure 3b–d. The S2 p_3/2_ and S2 p_1/2_ states in Figure 3b appeared at 162.8 and 164.2 eV binding energy, respectively. The Mo 3d_5/2_ and Mo 3d_3/2_ peaks in Figure 3c are located at 229.9 and 233 eV, respectively, and are attributed to the Mo^4+^ in MoS_2_ [34]. In addition, there was no Mo^6+^3d_3/2_ orbital peak at 236 eV in this spectra, indicating that the Mo element was not oxidized, but was converted to complete sulfide [35,36]. The W 4f spectrum in Figure 3d contains W 4f_7/2_, W 4f_5/2_, and W 5p_3/2_ peaks with binding energies at 33.4, 35.5, and 39.6 eV, respectively [37]. Colors that appearing in subfigures b–d are just to differentiate different fit peaks. The aforementioned results indicate that the 5 MW composite comprises the elements Mo, W, and S, implying the successful formation of this composite.

In order to determine the valence band positions of MoS_2_ and WS_2_, first we assessed the Mott-Schottky curves of the materials to determine the positions of the Fermi energy levels, and the energies between the valence bands to the Fermi energy levels were determined by VB-XPS [38]. The XPS valence band scan depicted in Figure 3e,f revealed that the energy gaps between the maximum valence band (VBM) and the Fermi energy levels are 1.04 eV for MoS_2_ and 1.03 eV for WS_2_, respectively. The dashed lines in Figures e,f are background fit lines of VB-XPS, which is to obtain the energy between valence band and the Fermi level.

**Figure 4:**
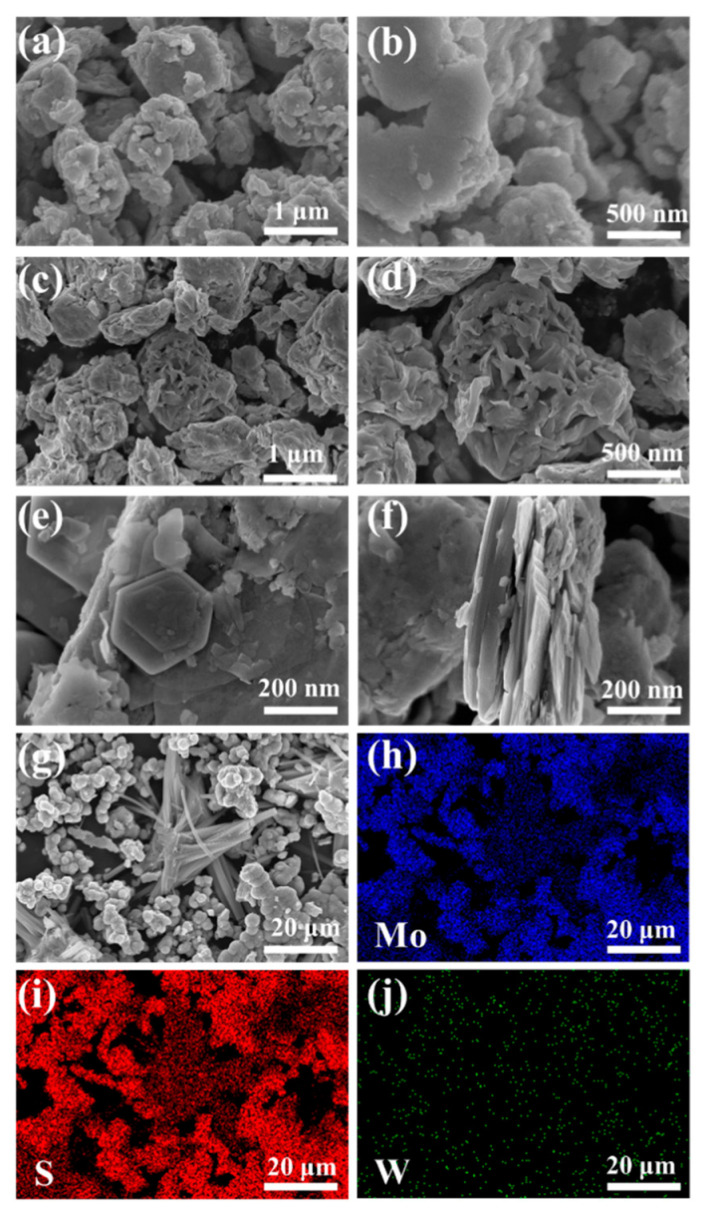
The SEM images of (**a**,**b**) WS_2_. (**c**,**d**) MoS_2_, and (**e**–**g**) 5 MW. (**h**–**j**) The elemental mapping of the 5 MW sample.

SEM images of WS_2_ in Figure 4a,b display a flake morphology with a thick and aggregated structure containing several grains attached to the surface. Figure 4c,d demonstrates that compared to the WS_2_ samples, MoS_2_ samples were less agglomerated and exhibited a flower-like morphology. Figure 4e–g displays the surface morphology of the 5 MW composite at different magnifications; the flower-like morphology can be observed more clearly in Figure 4g. Under high magnification SEM, the composite material displayed a lamellar structure. Figure 4f reveals that the 5 MW composite with a more uniform distribution exhibited a multilayer structure similar to that of the WS_2_ flakes, thereby providing more active sites for the desired gas absorption [39]. Figure 4h–j depicts the elemental mapping of the 5 MW composite, with a selected area shown in Figure 4g. In the 5 MW composites, S, Mo, and W were uniformly distributed, and no other elements were found.

**Figure 5:**
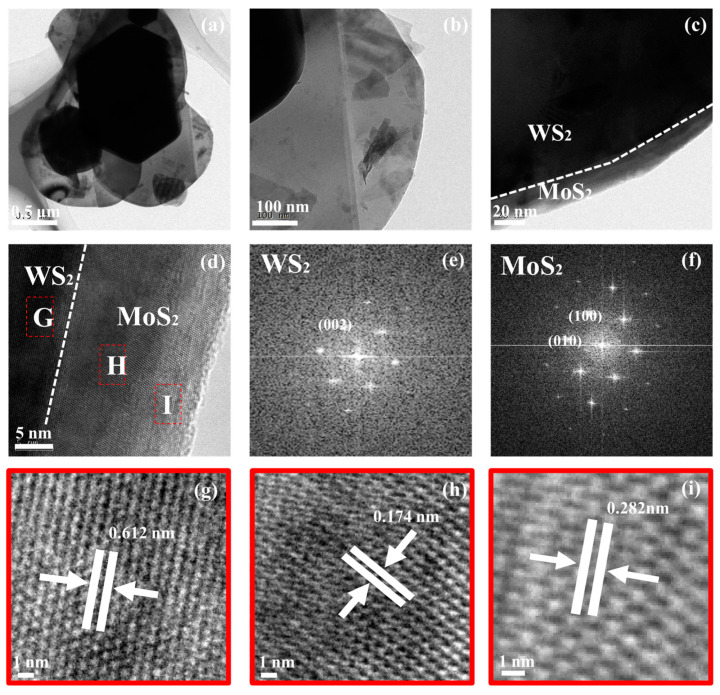
TEM images (**a**–**d**) and HRTEM images (**g**–**i**) of the 5 MW nanosheets; (**e**,**f**) are the FFT diffraction patterns of the dashed boxes (G,H region in (**d**)), which corresponds to the MoS_2_ and WS_2_ components, respectively.

The formation of MoS_2_/WS_2_ heterogeneous structures was confirmed using TEM. Figure 5a,b displays the 5 MW composite with a distinct ductile laminate structure. Figure 5c reveals a low-magnification TEM image of the heterostructure, where the light contrast outer layer was MoS_2_, while the inner layer was WS_2_. In addition, the magnified TEM image in Figure 5d depicts the interface between WS_2_ and MoS_2_, in which G, H, I are selected regions to progress further HRTEM analysis. Figure 5e,f represent the fast Fourier transform (FFT) patterns generated from the MoS_2_ and WS_2_, respectively (regions G and H in Figure 5). The same orientation and alignment of these diffraction points indicate an epitaxial relationship existing between MoS_2_ and WS_2_. However, the thickness of the MoS_2_ varies with the amount of MoS_2_ precursors utilized during the synthesis. HRTEM images of Figure 5g–i corresponds to the region depicted in the (G,H,I) region of Figure 5d, where uniform lattices with a spacing of 0.174 nm and 0.282 nm are observed, corresponding to the (100) and (010) planes of MoS_2_. The dotted stripe of 0.612 nm corresponds to the WS_2_ (002) plane (Figure 5e). The clear interface observed between WS_2_ and MoS_2_ in Figure 5d indicates a semiconductor heterojunction structure formation. The semiconductor heterojunction structure improves the electron-hole separation and transport properties, thereby enhancing the response speed of the gas sensors [40].

**Figure 6:**
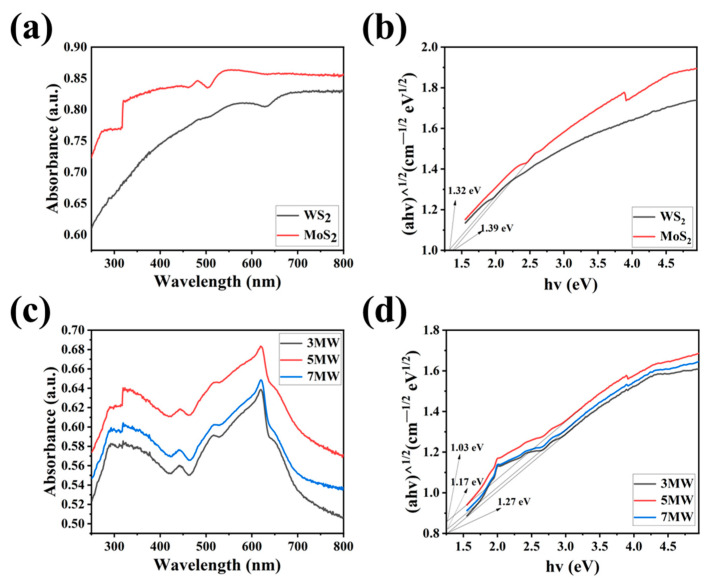
(**a**,**c**) The UV-Vis absorption spectra of the WS_2_, MoS_2_, 3 MW, 5 MW, and 7 MW samples. (**b**,**d**) Tauc plots showing the band gaps of the WS_2_, MoS_2_, 3 MW, 5 MW, and 7 MW samples.

The UV-Vis absorption spectra and Tauc plots of the WS_2_, MoS_2_, 3 MW, 5 MW, and 7 MW samples are depicted in Figure 6a–d. The MoS_2_ and 3 MW, 5 MW, and 7 MW samples jumped at 320 nm due to the inhomogeneous surface of MoS_2_, but this effect is very weak for the material forbidden band width. The composite samples exhibited strong light absorption near 310–600 nm in the visible and ultraviolet spectra. It is evident that increasing the MoS_2_ content improves the absorption of the 3 MW, 5 MW, and 7 MW samples in the 400–760 nm range. Furthermore, the absorption edge of WS_2_ shifted to the visible region (500–700 nm) after modification by MoS_2_, which may be caused by the coupling band between WS_2_ and MoS_2_ and leads to a reduction in the exciton recombination time [41]. Figure 6b,d demonstrate the energy band gaps for the samples; the energy band gap E_g_ was calculated using the well-known Tauc plot. The calculation is performed as follows [42]:

(αhv)^1/n^ = A(hv − E_g_)

α = 4πk/λ

where hv, α, k, and λ represent the photoelectron energy, absorption coefficient, absorption index, and wavelength of the photon, respectively, while n denotes the semiconductor type. In this work, the indirect transition was allowed [43]; therefore, n = 2. The calculated bandgaps of MoS_2_ and WS_2_ were 1.32 and 1.39 eV, respectively. It can also be determined according to the absorption edge, which was consistent with the bandgap calculated through the tangent. The bandgap of MoS_2_/WS_2_ composites became narrower as the MoS_2_ content increased, which facilitates the excitation of electrons from the valence band to the conduction band. 

**Figure 7:**
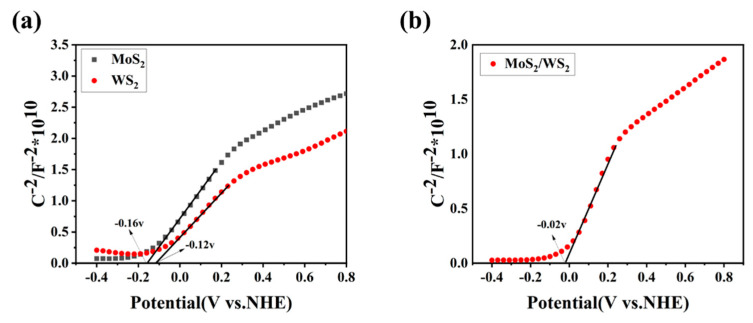
(**a**,**b**) The Mott-Schottky curves for the MoS_2_, WS_2_, and 5 MW samples.

The capacitance–voltage (C-V) relationship curves of the samples were measured with an electrochemical workstation (CHI-660E). The Mott-Schottky (M-S) curve was plotted with C^−2^ as the vertical axis and swept voltage as the horizontal axis. The slope of the most extended straight-line part of the curve determines the type of conductivity of the semiconductor [44]. Figure 7 depicts the Mott-Schottky curves for the pure-phase WS_2_, MoS_2_, and MoS_2_/WS_2_ composite solid powder samples. The Mott-Schottky slopes for the WS_2_ and MoS_2_ samples were positive, indicating that they are n-type semiconductors. The flat-band potentials (Fermi energy levels) were determined to be −0.16 V and −0.12 V, respectively (vs. NHE, pH = 7), by extending the linear part of the Mott-Schottky curve to the potential axis. The Mott-Schottky slope was also found to be positive in the MoS_2_/WS_2_ composite, indicating that it is an n-type semiconductor, and the flat-band potential (Fermi energy level) was −0.02 V (vs. NHE, pH = 7). An n-n heterojunction at the MoS_2_ and WS_2_ contact interface was confirmed using TEM analysis. This is consistent with the measurement results of the gas-sensing properties. When the VB-XPS result was combined with the Fermi energy levels of −0.16 V and −0.12 V, determined by Mott-Schottky analysis, the valence band positions for MoS_2_ and WS_2_ were calculated to be 0.88 V and 0.91 V, respectively. According to the UV-Vis absorption spectra, the band gap of WS_2_ was 1.39 eV, and that of MoS_2_ was 1.32 eV, respectively. The conduction bands of MoS_2_ and WS_2_ were located at −0.44 V and −0.48 V, respectively.

**Figure 8:**
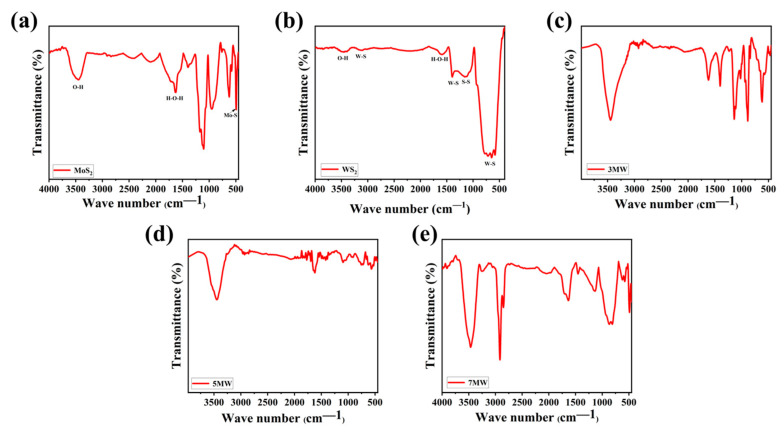
The FT-IR spectra of the (**a**) MoS_2_, (**b**) WS_2_, (**c**) 3 MW, (**d**) 5 MW, (**e**) 7 MW samples.

FT-IR spectral analysis reveals the surface chemical information and the vibrational modes of the chemical bonds present in the samples. Figure 8a–e depicts the IR spectra of MoS_2_, WS_2_, and their composite samples with varying relative mass ratios, revealing the surface functional groups. For WS_2_, the stretching vibrations of the W-S bond peak were located at approximately 2950 and 582 cm^−1^, respectively. Additionally, the S-S bond was at 1051 and 876 cm^−1^, respectively [45,46]. MoS_2_ exhibited typical Mo-S bond vibrations, with an absorption peak at 465 cm^−1^ [47]. The H-O-H bending motion and the O-H stretching vibrations of the adsorbed water on the surface of the composite were reflected at the peaks of 1626 and 3430 cm^−1^, respectively [48,49]. The broad peak at 3430 cm^−1^ was attributed to the symmetric and asymmetric O-H bond stretching patterns, which was more obvious in the composite material compared to the pure phases of WS_2_ and MOS_2_. This is advantageous for the detection of NH_3_, as the hydroxyl group has gas adsorption capacity, and improves the selectivity in the detection of NH_3_ due to the hydroxyl group's ability to hydrogen bond and weakly interact with ammonia, where the hydrogen molecules of ammonia combine with the oxygen molecules on the hydroxyl group to form a new chemical bond, thus allowing the ammonia to stay on the surface of the material for a longer period of time, which in turn improves the response of the material to NH_3_ [50]. The peaks of the W-S, S-S, Mo-S, O-H, and H-O-H bonds in MoS_2_/WS_2_ composites were derived from the MoS_2_ and WS_2_ components. Furthermore, the slight variation in the spectral shape (Figure 8c–e) indicates that the same groups were observed in different composite samples. The increased number of hydroxyl groups indicates that the composites possess better NH_3_ sensitivity properties than the pure phase.

**Figure 9:**
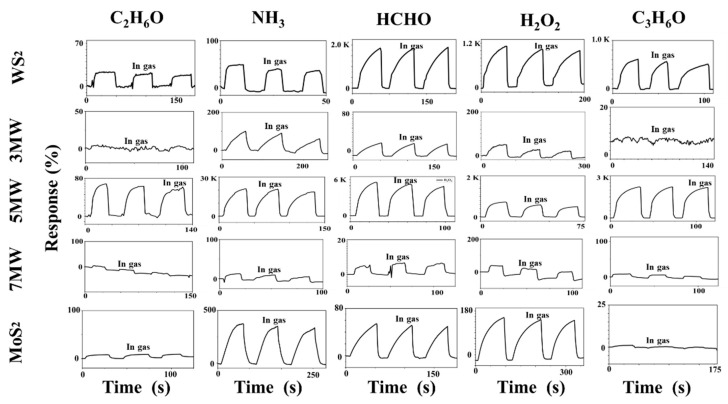
Responses of MoS_2_, WS_2_, and their composites to different gases (C_2_H_6_O, NH_3_, HCHO, H_2_O_2_, and C_3_H_6_O, respectively) of 500 ppm, 30% RH at room temperature.

The dynamic responses of the WS_2_, MoS_2_, and MoS_2_/WS_2_ composites to formaldehyde (HCHO), ethanol (C_2_H_6_O), ammonia (NH_3_), acetone (C_3_H_6_O), and hydrogen peroxide (H_2_O_2_) at room temperature are illustrated in Figure 9. The baseline current corresponds to an ambient test curve at approximately 30% RH (relative air humidity). The gas-sensing properties were obtained after three complete testing cycles, with the concentrations of all gases having been set to 500 ppm. Figure 9 demonstrates that the current response of the gas sensor increased rapidly when exposed to the vapors of HCHO, C_2_H_6_O, NH_3_, C_3_H_6_O, and H_2_O_2_. The response time is the time required to reach 90% of the maximum response current when the sensor is placed in the target gas, whereas the recovery is defined as the period of time when the sensor current changes to 10% of the response after removing the target gas. The response recovery curve in the figure reflects the n-type semiconductor’s characteristics. In our tests we found that MoS_2_ sometimes shows a negative response. This is because MoS_2_ is an n-type semiconductor, which is inevitably disturbed by ambient humidity during the performance test, and the adsorption of water molecules on the MoS_2_ surface equates to a p-type doping. As a result, the electrical properties change accordingly, and thus these samples show a response similar to the p-type semiconductor-based gas sensor in the reducing gas [51]. The responses of the pristine MoS_2_ and WS_2_-based sensors to NH_3_ were approximately 320% and 90%, respectively; while both WS_2_ and MoS_2_ exhibited a low NH_3_ response. The performance of composite-based sensors to HCHO, C_2_H_6_O, NH_3_, and C_3_H_6_O was also investigated at room temperature. The 5 MW samples outperformed WS_2_ and MoS_2_ in terms of detection performance to 500 ppm NH_3_ at a test voltage of 1 V, with a response of 23643%, enhanced by two orders of magnitude. Additionally, the 5 MW samples exhibited 2200%, 2310%, 672%, and 120% responses for other gases such as H_2_O_2_, C_3_H_6_O, HCHO, and C_2_H_6_O, respectively, indicating a good selectivity for the detection of targeted NH_3_ gases. The recovery time of the composite samples was particularly fast, with a relatively long response time for the detection of these five gases. The 5 MW composite exhibited a response time of 30 s and a recovery time of 2.6 s. The 5 MW samples demonstrated a good reproducibility for NH_3_ detection after consecutive test cycles. These results reveal that the prepared 5 MW samples are excellent for NH_3_ detection at room temperature, and that the introduction of composite components improves the gas-sensing properties of pristine WS_2_ or MoS_2_ samples.

**Figure 10:**
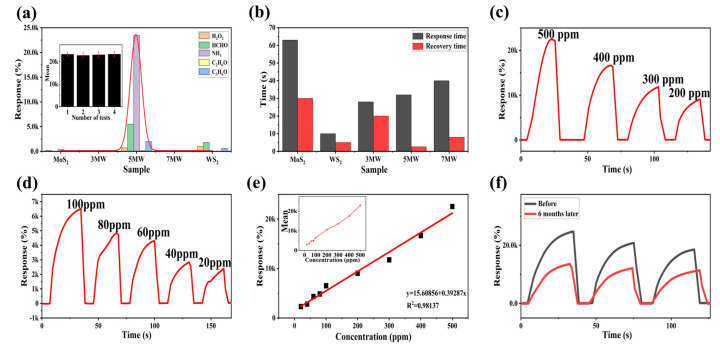
(**a**) Selectivity of MoS_2_, WS_2_, and composite samples for different gases of 500 ppm at room temperature. (**b**) Response and recovery time of the 5 MW-based gas sensor to 500 ppm NH_3_ at room temperature. (**c**,**d**) Responses of the 5 MW sample to different concentrations of NH_3_ at room temperature. (**e**) Linear response of the 5 MW sensor to varying concentrations of NH_3_. (**f**) Long-term stability of the 5 MW sensor for six months.

The selectivity plots of WS_2_, MoS_2_ and MoS_2_/WS_2_ composites for HCHO, C_2_H_6_O, NH_3_, C_3_H_6_O, and H_2_O_2_ gases are depicted in Figure 10a. When compared to other composite samples, the response of the 5 MW sample to NH_3_ reached nearly four orders of magnitude higher, demonstrating that the 5 MW samples exhibit a high selectivity for NH_3_ detection. The histogram of response/recovery time is shown in Figure 10b. Compared with other samples, the 5 MW composite had a response time of about 30 s and a recovery time of only 2.6 s, revealing an efficient detection performance and a fast recovery for NH_3_ detection. Figure 10c,d depicts the response versus different concentrations of NH_3_ at room temperature. The current response of the 5 MW gas sensor increases with increasing NH_3_ concentration, which can be attributed to the increased possibility of gas adsorption at the active sites on the sample surface. In addition, the 5 MW gas sensor exhibits a highly linear relationship for NH_3_ detection in the concentration range of 20–500 ppm. The linear relation was as follows: R = 0.39287C + 15.60856 (R^2^ = 0.98137 in Figure 10e). Figure 10f exhibits a six-month response comparison of the 5 MW sensor to 500 ppm NH_3_ at room temperature. The response reaches four orders of magnitude both before and after six months, confirming the excellent long-term stability of the 5 MW gas sensor.

**Figure 11:**
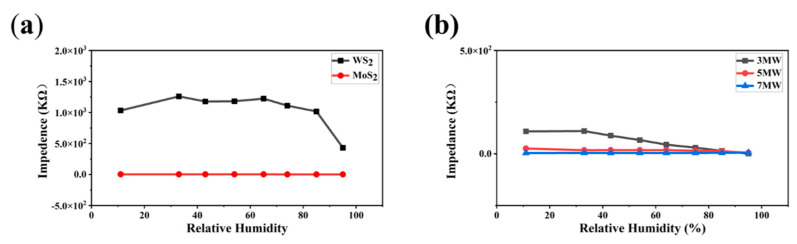
(**a**) Response curves of the MoS_2_, WS_2_ samples to 11–95% RH; (**b**) Response curves of the MoS_2_/WS_2_ composites to 11–95% RH.

Humidity immunity tests were performed to eliminate humidity interference with the gas sensor. The prepared sensors were placed in chambers with different humidity levels (11–95% RH) by rapidly switching the sensors from one chamber to another. Impedance versus humidity curves for the pristine MoS_2_ and WS_2_ at 100 Hz, AC 1 V are displayed in Figure 11a. The response was less than one order of magnitude for MoS_2_ and one order of magnitude for WS_2_ over the humidity range of 11–95% RH. Figure 11b reveals that the 5 MW composite exhibited excellent humidity immunity, and the moisture-sensitive response was less than an order of magnitude. These results indicate that both the pure-phase and composite samples had a high humidity resistance. 

Table 1 summarizes the performance of NH_3_ sensors reported in the literature in recent years. Compared to the previous studies, the 5 MW composite prepared in this work exhibits a shorter recovery time (2.6 s), and a higher response of 23643% to 500 ppm NH_3_.

## 4. Discussion

**Figure 12:**
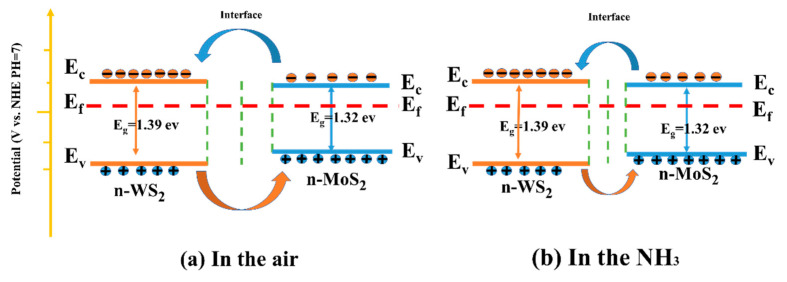
The sensing mechanism of the MoS_2_/ WS_2_ gas sensor in (**a**) air and (**b**) NH_3_.

The energy band was determined by combining the Mott-Schottky curve, XPS valence band spectrum, and UV-visible absorption spectrum, as illustrated in Figure 12. As demonstrated through the previous analysis, the slopes of Mott-Schottky curves of WS_2_, MoS_2_ and MoS_2_/WS_2_ samples were all positive, implying that the prepared samples are n-type semiconductors, while the TEM analysis demonstrated that heterostructures were formed between the two components. In particular, n-n-type MoS_2_/WS_2_ heterostructures were developed in this study, which are commensurate with the adsorption–desorption curve characteristics of WS_2_, MoS_2_, and MoS_2_/WS_2_ sensors. The electrons would be transferred between MoS_2_ and WS_2_ until the Fermi energy level reaches equilibrium.

The NH_3_ gas-sensitive mechanism of the MoS_2_/WS_2_ sensor was explained using a surface resistance control model, which included the change in the sensor resistance due to the redox reaction between the chemisorbed oxygen and the target gas molecules [57]. When the MoS_2_/WS_2_ composite was exposed to air at room temperature, O_2_ molecules were adsorbed on the sample surface, and free electrons on the composite surface were captured by O_2_ to produce surface adsorbed species (O^2−^, O^−^, O_2_^−^), as illustrated in Figure 12a. The corresponding reaction processes are described in Equations (1) and (2) [58].

O_2_ (gas) → O_2_ (adsorption) (1)

O_2_ (adsorption) + e^−^ → O_2_^−^ (adsorption) (<100 °C) (2)

At this point, a wide depletion layer has been formed on the sample surface due to a severe lack of electrons, while the sensor’s resistance is high. When the MoS_2_/WS_2_ composite is exposed to NH_3_, the reducing gas interacts with the adsorbed O^2−^ to produce H_2_O, N_2_, and free electrons, according to Equations (3) and (4).

NH_3_ (gas) ↔ NH_3_ (adsorption) (3)

4NH_3_ (adsorption) + 3O_2_^−^ (adsorption) → 6H_2_O + 2N_2_ + 3e^−^ (4)

The captured electrons are subsequently returned to the sample surface. This process significantly reduces the thickness of the electron depletion layer. As a result, the electron depletion layer narrows, and the resistance reduces (Figure 12b).

The development of the heterojunction was deemed to be the main reason for the enhanced gas-sensitive response. As previously demonstrated, WS_2_ and MoS_2_ are n-type semiconductors with electrons as the main charge carriers. The Fermi energy level of MoS_2_ was higher than that of WS_2_, as illustrated by the Mott-Schottky curves. Electrons would be transported in the air from MoS_2_ to WS_2_ until the Fermi energy levels reached equilibrium. In contrast, carriers (holes) leaving the valence band would diffuse in the opposite direction to achieve effective charge-carrier separation. Consequently, the energy band bends, creating a barrier that impedes electron transport. Therefore, the baseline resistance of the WS_2_/MoS_2_ sensors significantly increased. The resistance was further reduced when the sensor was placed in NH_3_ since the electrons released by the reaction with O_2_^−^ lowered the potential barrier in the heterojunction region. Therefore, the sensing performance of MoS_2_/WS_2_ composites to NH_3_ was enhanced due to the substantial change in device resistance [59,60].

On the other hand, the superior sensing performance of the MoS_2_/WS_2_ composites over pristine WS_2_ and MoS_2_ can be attributed to the flower-like morphology of the WS_2_/MoS_2_ composites compared to the homogeneous sheet-like morphology of pure-phase WS_2_ and MoS_2_, which provides more active sites for surface reactions and promotes gas adsorption and desorption, thus improving the gas-sensing response.

In short, the excellent gas-sensing properties of MoS_2_/WS_2_ composites are attributed to the gain effect of the n-n heterojunction and unique surface morphology.

## 5. Conclusions

In this study, MoS_2_/WS_2_ composites were prepared using a one-step hydrothermal method that is highly adaptable, affordable, and accessible. A heterostructure was successfully formed by controlling the relative mass ratio of WS_2_ and MoS_2_, revealing carrier movement and transformation at the MoS_2_/WS_2_ interface. The positions of the valence and conduction bands of the two-component semiconductors were determined by the Mott-Schottky curves, UV-Vis spectra, and XPS valence band spectra. Afterward, WS_2_-MoS_2_ heterojunction-based gas-sensitive sensors were fabricated. The 5 MW sample exhibited good gas-sensitive characteristics when the relative mass ratio of MoS_2_ and WS_2_ was 50 wt% with a response of 23643% for 500 ppm NH_3_ gas, a fast recovery time of 2.6 s, a detection limit of 20 ppm, and long-term stability for six months. The humidity immune test demonstrated that the 5 MW sample possesses good moisture resistance in the humidity range of 11–95% RH. This work provides an approach for the simple and low-cost preparation of two-dimensional gas-sensing materials with heterostructures. It also provides a facile way to obtain NH_3_ gas sensors working at room temperature.

## Figures and Tables

**Table 1:** Comparison of sensing parameters of the 5 MW sensor with previously reported sensors.

Materials	T (°C)	Concentration (ppm)	Response/Recovery Time (s)	Limit of Detection (ppm)	Gas Type	Response	Ref.
P-MoS_2_	150 °C	50	1300/1250	10	NH_3_	651.53%	[52]
Ti_3_C_2_Tx	RT	100	-	9.27	NH_3_	21%	[53]
C-xy graphene	RT	500	-	-	NH_3_	4200%	[54]
Pt-Ti_3_C_2_Tx/TiO_2_	RT	100	23/24	10	NH_3_	45.5%	[55]
Ti_3_C_2_Tx/Ti_3_AlC_2_	RT	0.5	90/75	0.05	NH_3_	1.2%	[56]
MoS_2_/WS_2_	RT	500	30/2.6	20	NH_3_	23643%	This work

## Data Availability

The data presented in this study are available on request from the corresponding author.
